# Blood Monocyte Phenotype Fingerprint of Stable Coronary Artery Disease: A Cross-Sectional Substudy of SMARTool Clinical Trial

**DOI:** 10.1155/2020/8748934

**Published:** 2020-07-27

**Authors:** Silverio Sbrana, Jonica Campolo, Alberto Clemente, Luca Bastiani, Antonella Cecchettini, Elisa Ceccherini, Chiara Caselli, Danilo Neglia, Oberdan Parodi, Dante Chiappino, Jeff M. Smit, Arthur J. Scholte, Gualtiero Pelosi, Silvia Rocchiccioli

**Affiliations:** ^1^CNR Institute of Clinical Physiology, 54100 Massa, Italy; ^2^CNR Institute of Clinical Physiology, 20162 Milan, Italy; ^3^Fondazione Toscana Gabriele Monasterio, 56124 Pisa, Italy; ^4^Department of Clinical and Experimental Medicine, University of Pisa, 56126 Pisa, Italy; ^5^CNR Institute of Clinical Physiology, 56124 Pisa, Italy; ^6^Department of Cardiology, Leiden University Medical Center, 2333 ZA, Leiden, Netherlands

## Abstract

**Background and Aims:**

Atherosclerosis is an inflammatory disease with long-lasting activation of innate immunity and monocytes are the main blood cellular effectors. We aimed to investigate monocyte phenotype (subset fraction and marker expression) at different stages of coronary atherosclerosis in stable coronary artery disease (CAD) patients.

**Methods:**

73 patients with chronic coronary syndrome were evaluated by CT coronary angiography (CTCA) and classified by maximal diameter stenosis of major vessels into three groups of CAD severity: CAD1 (no CAD/minimal CAD, *n*° = 30), CAD2 (non-obstructive CAD, *n*° = 21), and CAD3 (obstructive CAD, *n*° = 22). Flow cytometry for CD14, CD16, and CCR2 was used to quantify Mon1, Mon2, and Mon3 subsets. Expression of CD14, CD16, CD18, CD11b, HLA-DR, CD163, CCR2, CCR5, CX3CR1, and CXCR4 was also measured. Adhesion molecules and cytokines were quantified by ELISA.

**Results:**

Total cell count and fraction of Mon2 were higher in CAD2 and CAD3 compared to CAD1. By multivariate regression analysis, Mon2 cell fraction and Mon2 expression of CX3CR1, CD18, and CD16 showed a statistically significant and independent increase, parallel to stenosis severity, from CAD1 to CAD2 and CAD3 groups. A similar trend was also present for CX3CR1 and HLA-DR expressions on total monocyte population. A less calcified plaque composition was associated to a higher Mon2 expression of CD16 and higher TNF-*α* levels. IL-10 levels were lower at greater stenosis severity, while the IFN-*γ*/IL-10 ratio, a marker of a systemic pro-inflammatory imbalance, was directly correlated to stenosis degree and number of noncalcified plaques.

**Conclusions:**

The results of this study suggest that a specific pattern of inflammation-correlated monocyte marker expression is associated to higher stenosis severity and less calcified lesions in stable CAD. The clinical trial Identifier is NCT04448691.

## 1. Introduction

Atherosclerosis is a chronic non-resolving inflammatory disease of the vessel wall, characterized by the long-term activation of the innate immune system [1, 2].

Blood monocytes and tissue-resident macrophages are the main cellular effectors of innate immunity. Monocyte accumulation within the vascular wall, in particular, contributes to atherogenesis and plaque growth through the generation of “foam” macrophages and the promotion of further leukocyte recruitment into complicated plaques [3, 4]. Following the discovery of blood monocyte subsets, CD14 and CD16 have emerged as standard markers of their definition [5]. The 2010 nomenclature document, in accordance with the recommendation of the Nomenclature Committee of the International Union of Immunological Societies, acknowledged the existence of three main circulating subsets and standardized their definitions as CD14^++^CD16^−^ or “classical,” CD14^++^CD16^+^or “intermediate,” and CD14^+^CD16^++^ or “non-classical” [6].

It has been demonstrated that innate immune cells can adopt a persistent pro-inflammatory functional phenotype after exposure to a variety of activating stimuli, defined as “trained innate immunity” [7]. The idea that trained innate immunity contributes to the development and progression of atherosclerosis is progressively emerging, both in the setting of traditional cardiovascular risk factors and in the setting of non-traditional risk factors, such as acute and chronic infections, as well as non-infectious chronic inflammatory disorders [8–10]. A pathophysiological relation between atherosclerosis extent, plaque composition, and the circulating mononuclear phagocyte system profile is therefore hypothesized. Previous studies, performed in large prospective cohorts, revealed that an increase of intermediate CD14++/CD16+ monocytes in the blood is an independent predictor of acute adverse cardiovascular outcomes in subjects with chronic kidney disease, presence of many cardiovascular risk factors, and after percutaneous transluminal coronary angioplasty (PTCA) [11–13]. However, few studies have investigated the relationship between the phenotype of blood monocytes and the stenosis severity of coronary artery disease (CAD), assessed by non-invasive CTCA coronary imaging in stable patients with chronic coronary syndrome according to ESC definition [14].

Accordingly, this study is aimed at assessing blood monocyte subset distribution and functional phenotype marker expression at different stages of CAD—classified by guidelines recommended for the standard criteria, based on the degree of maximal diameter stenosis in major vessels—as a putative fingerprint of the presence of an innate immunity training process.

For this purpose, in an observational cross-sectional substudy of 73 patients with chronic coronary syndrome, prospectively included in the clinical study of the EU Project SMARTool (GA number: 689068) to receive a computed tomography coronary angiography (CTCA) scan [15], we analyzed blood monocyte phenotypic profile in three groups of patients, classified according to the standard stenosis criteria, from minimal to non-obstructive and obstructive diseases.

## 2. Materials and Methods

### 2.1. Patients

The study was approved by the Ethical Committee of the “Area Vasta Nord-Ovest” (CEAVNO Prot. number 23534) of Tuscany Region (Italy) and was conducted in accordance with the Declaration of Helsinki, and all patients provided written informed consent. Patients (*n*° = 73) were recruited at the Cardiology and Cardiovascular Medicine Division of Fondazione Toscana “G. Monasterio” (Pisa–Italy) from September 2016 to November 2017, within the H2020 EU Project SMARTool. Patients in stable clinical conditions and referred to CTCA for suspected stable CAD were included according to the study criteria reported in Supplementary Materials (see Table S1).

### 2.2. Computed Tomography Coronary Angiography (CTCA) Image Acquisition Protocol

Subjects underwent ECG-triggered cardiac CTCA scan using 64-slice scanners or higher, according to the predefined standard operating procedure of SMARTool Project Clinical Study, in order to ensure optimal image quality. The main points of the standard operating procedure (SOP) for CT scan acquisition, relevant for accurate analysis, can be summarized as follows:

- Absence of motion or other artifacts in acquired images
- Heart rate < 65 beats/min.
- Nitroglycerin administration prior to acquisition.
- Optimized reconstruction of the most suitable cardiac cycle (diastole at 70%-80% RR interval).
- Upload of multiple cardiac phases as to identify all coronary segments if needed.
- Reconstructed field of view of 200-250 mm for CTCA.

All coronary CTCA images were analyzed blinded to clinical data in a Core Laboratory (Leiden University Medical Center), and coronary arteries were visually analyzed according to the modified 17-segment American Heart Association classification [16].

CAD was assessed by semi-quantitative visual assessment of two expert cardioradiologists and classified by maximal diameter stenosis percentage in at least 1 of major epicardial artery segments into the following severity classes, in accordance with computed tomography (CT) CAD-RADS guidelines [17, 18]: Class 0: no plaque nor stenosis in any major vessel (*n*° = 14); Class 1: stenosis < 25% (minimal CAD) (*n*° = 16); Class 2: 25% ≥ stenosis < 50% (non-obstructive CAD) (*n*° = 21); Class 3: 50% ≥ stenosis < 70% (sub-obstructive CAD) (*n*° = 14); and Class 4: stenosis ≥ 70% (obstructive CAD) (*n*° = 8). The five CAD-RADS classes have been merged into three main categories of stenosis severity, in agreement with the clinically oriented approach proposed by others [19]: CAD-RADS 0 or 1, CAD-RADS 2, and CAD-RADS 3 or 4, thus originating three subsets of patients: (a) no CAD/minimal CAD (CAD1), (b) non-obstructive CAD (CAD2), and (c) obstructive CAD (CAD3).

In each coronary artery segment, an atherosclerotic plaque is defined as a tissue structure above 1 mm^2^ on the cross-sectional view of the vessel wall. Mean plaque composition was qualitatively assessed as noncalcified, mixed, or calcified by using both fixed (-30 to 130, 131 to 350 and ≥351 HU density value, respectively) and adaptive, luminal contrast density-corrected, thresholds, as reported [20–22].

CAD staging by maximal stenosis degree is also representative, in our study population, of the extension of the atherosclerotic disease throughout the coronary tree, expressed as the total number of visible plaques at CTCA scan (see Table S2 in Supplementary Materials).

### 2.3. Biochemical Analyses

Peripheral blood samples were collected in Vacutainer® tubes for plasma and serum separation. EDTA-anticoagulated blood was centrifuged at 1000x*g*, for 10 minutes at 4°C in order to obtain plasma aliquots. Whole blood samples, collected in serum separator tubes, were kept at room temperature for 30 minutes to allow sample coagulation before centrifugation at 1000x*g*, for 20 minutes at 4°C. The supernatants (serum and plasma) were transferred into polypropylene tubes and stored in aliquots at -80°C. Blood cell count (*n*° of cells/*μ*l) was carried out by using the automated cell analyzer SF-3000 (Sysmex, Kobe, Japan). Serum lipid profile (total cholesterol, LDL and HDL cholesterol, and triglycerides) and general biochemical parameters (creatinine, uric acid, glucose, Hs-CRP, and fibrinogen) were measured according to routine clinical protocols. Antigenic immunoassay procedure based on enzyme-linked immunosorbent assay (ELISA) was instead used for the quantification of plasma adhesion molecules (VCAM-1, ICAM-1) and serum cytokines (IL-6, IFN-*γ*, TNF-*α*, and IL-10). The following commercial ELISA kits were used: DCD540 and DVC00 (R&D Systems, Inc.) for human ICAM-1 and VCAM-1 determinations; 950.035 (Diaclone) for high sensitivity IL-6 assessment; E-EL-H0108, E-EL-H0109 and E-EL-H0103 (Elabscience® Biotechnology) for IFN-*γ*, TNF-*α*, and IL-10 evaluations, respectively. All the biomarkers were determined in duplicates.

### 2.4. Flow Cytometry Analysis

**Figure 1:**
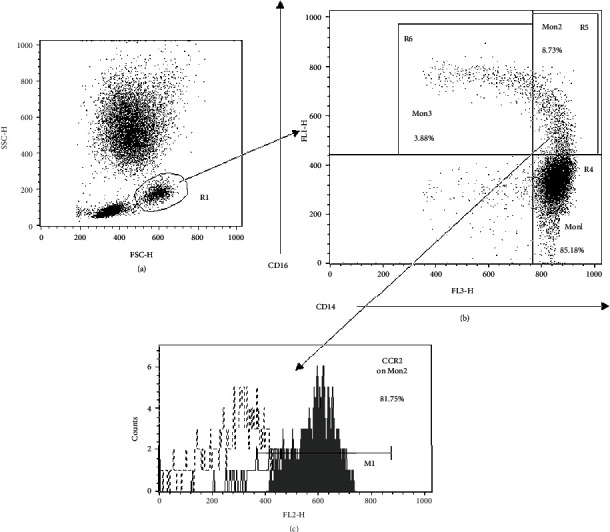
Representative example of the flow cytometry analysis for monocyte subset quantification. (a) Total monocyte cluster identification based on its forward and side scatter morphological characteristics (region R1). (b) Selection of CD14++/+ events and preliminary monocyte subset quantification (as percentage) [(CD14++/CD16- (Mon1, R4), CD14++/CD16+ (Mon2, R5), and CD14+/CD16++ (Mon3, R6)] initially based on their differential expression of markers CD14 and CD16. (c) The measurement of the actual circulating fraction of subsets Mon1 and Mon2 is obtained by multiplying, and then dividing by one hundred, the percentages of events measured at the point *b* (regions R4 and R5) with their corresponding percentages of positivity of the distinctive marker CCR2 (grey subtraction histogram by the Overton technique). For the final quantification of the Mon3 fraction (region R6), the value of its CCR2 negative fraction is used.

Flow cytometry was performed with a FACScan instrument interfaced with CellQuest software (Becton Dickinson, San Jose, CA, USA). Overtime monitoring of instrument alignment was carried out with CaliBRITE beads (Becton Dickinson). Flow cytometric identification and quantification of monocyte markers was performed on EDTA anticoagulated whole blood samples (50 *μ*l), within 1 hour after collection, by using a lyse-no-wash staining procedure, as described [23]. The following combinations of PC5- (Beckman Coulter), FITC- (BD Pharmingen) and PE-conjugated (R&D) mouse anti-human monoclonal antibodies were employed: (a) CD14/CD16/CCR2, (b) CD14/CD16/CCR5, (c) CD14/CD16/CX3CR1, (d) CD14/CD16/CXCR4, (e) CD14/CD16/CD18, (f) CD14/CD16/CD11b, (g) CD14/CD16/HLA-DR, and (h) CD14/CD16/CD163. Appropriate mouse isotype controls were carried out in parallel. In brief, after antibody incubation for 20 min at room temperature (RT) in the dark, erythrocytes were lysed with 750 *μ*l lysing buffer (BD Biosciences) for 10 min at RT before quenching with an equal volume of ice-cold PBS. The tubes were then maintained on ice until FACS analysis. The sequence of analysis, conducted according to the minimal requirement suggested by the joint consensus document of the European Society of Cardiology (ESC) Working Groups [24], is described below and represented in Figure 1: stringent monocyte morphological cluster identification based on its forward and side scatter characteristics [Figure 1(a)]; selection of CD14++/+ (bright/low) events and preliminary monocyte subsets quantification (as percentage) initially based on their differential expression of surface markers CD14 and CD16 (CD14++/CD16-, CD14++/CD16+, CD14+/CD16++) [Figure 1(b)]; measurement of the actual circulating fraction (percentage) of subsets Mon1(CD14++/CD16-/CCR2+) and Mon2(CD14++/CD16+/CCR2+) obtained by multiplying, and then dividing by one hundred, the percentages of subsets derived at the point *b* with their corresponding percentages of positivity for the distinctive marker CCR2 [Figure 1(c)]. These last are calculated by using an isotype median-based overlaid histogram subtraction analysis, according to the Overton subtraction technique [Figure 1(c)] [25]. For calculation of the actual percentage of subset Mon3 (CD14+/CD16++/CCR2-), the value of its CCR2 negative fraction was used.

The use of CCR2 is essential for determining the absolute count of the three monocyte subsets. The two markers CD14 and CD16 are enough for subset identification and characterization of their surface marker profile [23]. The expression of surface markers has been also quantified on the whole CD14++/+ monocyte population. The Overton electronic analysis allows the quantification of marker expression either in terms of percentage of positivity (%+) or relative fluorescence intensity (RFI) (median of the positive events distribution minus median of the isotype control histogram), as reported in our previous works [26–28].

### 2.5. Statistical Analysis

Continuous data are presented as mean ± mean standard error (SEM) and categorical variables as number of patients and percentage. The comparison between groups has been performed by ANOVA (with Bonferroni's correction) for continuous data and by Chi-Square (*χ*^2^) test for categorical data. At univariate and multivariate multinomial logistic regression analyses, the three categories of CAD stenosis severity have been considered the dependent variable and analyzed on the basis of the following grouping of variations: CAD1 vs. CAD2 (CAD1/CAD2) and CAD1 vs. CAD3 (CAD1/CAD3). Monocyte data significantly higher in higher stenosis severity groups by univariate logistic regression have been subsequently tested by multivariate logistic regression. Only monocyte data showing statistical significance in both groups of variation at multivariate logistic regression analysis have been considered. The variables used for the adjustment in multivariate logistic regression were continuous and categorical and were chosen by their known pathophysiological relevance in the development and progression of atherosclerotic disease. They included Framingham Risk Score, metabolic syndrome and diabetes, systemic pro-/anti-inflammatory environment (plasma levels of Hs-CRP and IL-6, IFN-*γ*, TNF-*α*, and IL-10), endothelial activation (plasma levels of ICAM-1 and VCAM-1), presence and dosage (mg/die) of statin therapy, and use of oral hypoglycemic drugs. Linear regression analyses have been performed to correlate monocyte activation markers HLA-DR on CD14++/+ population with monocyte subset frequencies. All statistical analyses were performed by StatView 5.0 software program (SAS Institute, Cary, NC, USA). A *P* value < 0.05 was considered statistically significant.

## 3. Results

### 3.1. Patients Characteristics

Patient demographic, clinical, and laboratory characteristics are reported in Table 1, as a whole and by the three groups of CAD.

Among CAD groups, males were more numerous in CAD3 (86.3%), when compared to CAD1 (56.7%) and CAD2 (57.1%). The frequency of diabetic patients increased progressively from CAD1 to CAD3. Total and LDL cholesterol concentrations were significantly lower in CAD3 patients compared to CAD1 and CAD2, probably as a consequence of the higher frequency of statin-treated subjects in this group (95.4%). Not only the use of statin therapy but also its daily dosage was significantly higher both in CAD2 and CAD3 compared to CAD1 patients. CAD2 patients had lower plasmatic levels of VCAM-1 when compared to other two, while no differences were observed in ICAM-1 concentrations among groups.

### 3.2. Cytokines

**Figure 2:**
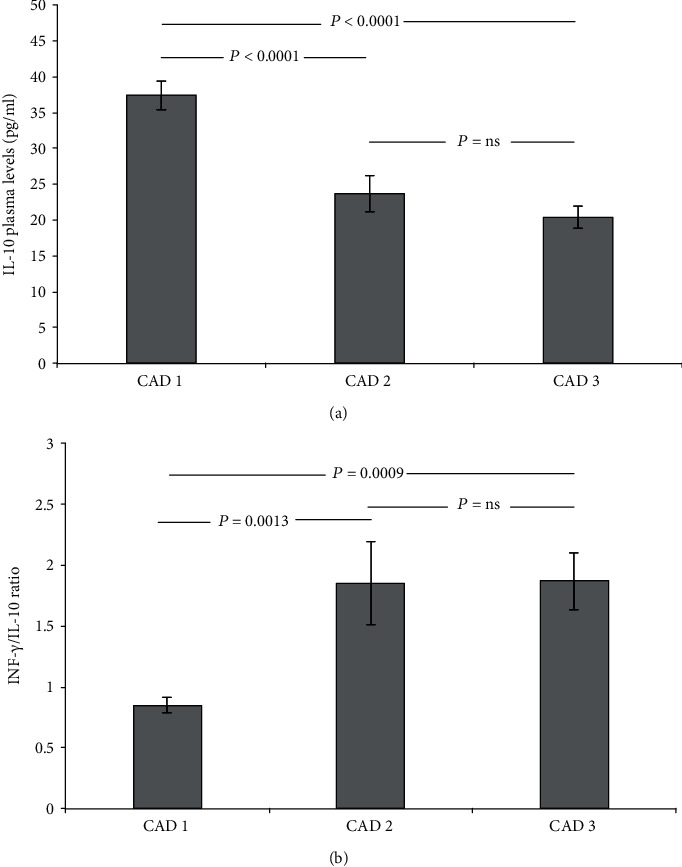
Cumulative graphic representation (mean ± SEM) of IL-10 plasma levels (pg/ml) [(a); ANOVA *P* < 0.0001] and IFN-*γ*/IL-10 ratio [(b); ANOVA *P* = 0.0006] in the three CAD severity groups. CAD1: no CAD/minimal CAD; CAD2: non-obstructive CAD; CAD3: obstructive CAD.

The ANOVA post-hoc test showed significant lower levels of IL-10 in CAD2 and CAD3 groups than in CAD1 [Figure 2(a)], together with a parallel increase of the IFN-*γ*/IL-10 ratio [Figure 2(b)], suggestive of a systemic pro-inflammatory unbalance. On the other hand, no significant differences were found in the plasma concentration of the other cytokines tested (VCAM-1, ICAM-1, IL-6, and TNF-*α*) relative to stenosis severity.

### 3.3. Monocyte Phenotype Analysis

The total cell count of CD14++/+ monocyte population was not significantly different in the three groups of CAD. However, by multivariate logistic regression, significantly higher RFI values were found in CAD2 and CAD3 groups for membrane receptors CX3CR1 (*P* = 0.0118, OR = 1.087, *CAD1/CAD2*; *P* = 0.0478, OR = 1.066, *CAD1/CAD3*) and HLA-DR (*P* = 0.0353, OR = 1.030, *CAD1/CAD2*; *P* = 0.0170, OR = 1.038, *CAD1/CAD3*).

**Figure 3:**
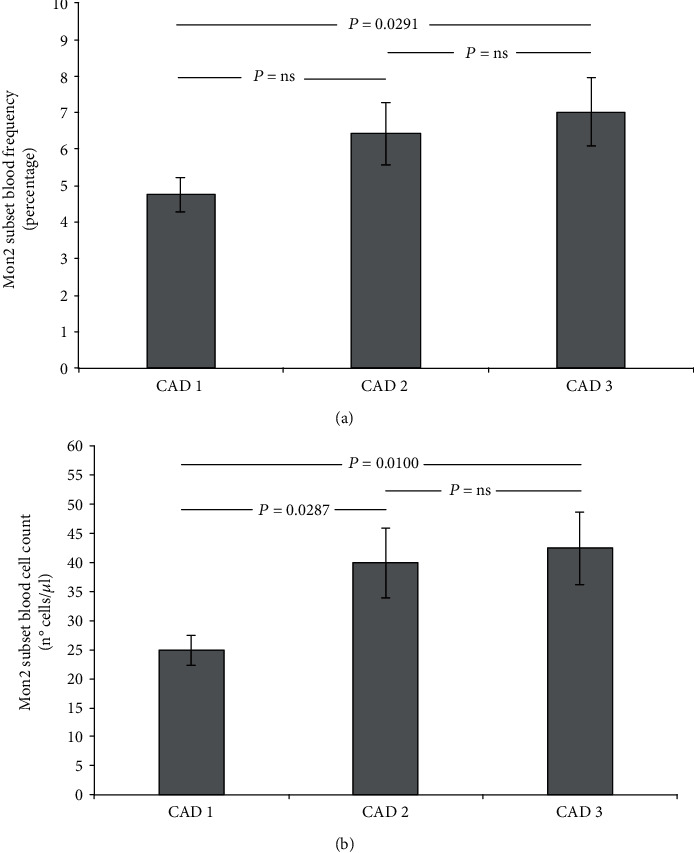
Cumulative graphic representation (*mean* ± *SEM*) of Mon2 subset circulating fractions [(a); ANOVA *P* = 0.0695] and counts [(b); ANOVA *P* = 0.0177] in the three CAD severity groups. CAD1: no CAD/minimal CAD; CAD2: non-obstructive CAD; CAD3: obstructive CAD.

Subset analysis evidenced higher values of Mon2 subset fraction in CAD2 (6.42 ± 0.85) and CAD3 (7.02 ± 0.94) compared to CAD1 (4.75 ± 0.47). Moreover, a significantly higher Mon2 cell count (n°. of cells/*μ*l) was also found in CAD2 (39.89 ± 5.98) and CAD3 (42.44 ± 6.26) compared to CAD1 (24.88 ± 2.58). Cumulative data, including statistical differences, of Mon2 circulating cell fraction and count from CAD1 to CAD3 are shown in Figures 3(a) and 3(b), respectively. The Mon3 subset cell count shows a statistically not significant trend to increase from CAD1 (38.02 ± 2.61) to CAD2 (46.33 ± 3.75) and CAD3 (48.18 ± 5.81).

Monocyte subset frequencies and counts, compared by univariate logistic regression and subsequently processed by multivariate logistic regression analysis in the three groups of CAD stenosis severity, are summarized in Table 2.

**Figure 4:**
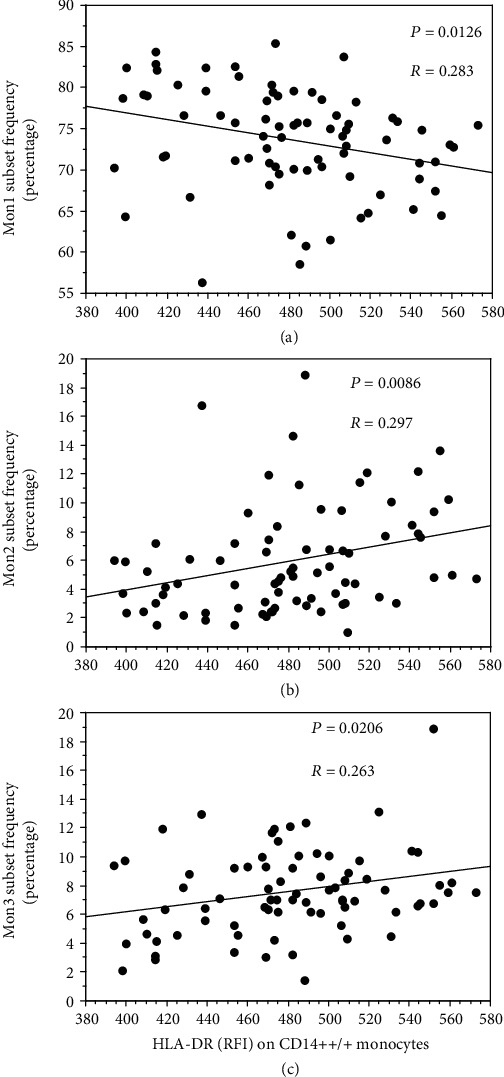
Linear correlations between the HLA-DR expression level (as RFI units) of all CD14++/+ monocytes and the circulating fractions (as percentages) of the Mon1 (a), Mon2 (b), and Mon3 (c) monocyte subsets.

Significant relationships were found between the circulating fractions of monocyte subsets and the expression level (as RFI) of the monocyte activation marker HLA-DR on all CD14++/+ cells. In particular, HLA-DR expression correlated positively with Mon2 [Figure 4(b)] and Mon3 [Figure 4(c)] frequencies, and negatively with Mon1 frequency [Figure 4(a))]

Following multivariate logistic regression, Mon2 cell fraction was the only subset showing a significant progressive increase from CAD1 to CAD3 (Table 2), and the same significant trend was present for Mon2 markers CX3CR1 and CD18, expressed as RFI, and CD16, evaluated as percentage of positivity (Table 3). No changes in Mon3 markers among the 3 groups were found, while in subset Mon1, the RFI expression level of HLA-DR was also higher in the CAD2 and CAD3 groups (*P* = 0.0425, OR = 1.031, *CAD1/CAD2*; *P* = 0.0281, OR =1.035, *CAD1/CAD3*).

### 3.4. Associations between Immunological Features and Plaque Composition

RFI values of CD16 on all circulating CD14++/+ monocytes showed a significant positive correlation with number of mixed plaques (*P* = 0.0024, *R* = 0.348) in the whole CAD population (73 patients). Mon2 RFI values of CD16 correlated both with the number of mixed plaques (*P* = 0.0023, *R* = 0.349) and with the cumulative number of noncalcified and mixed plaques (*P* = 0.0085, *R* = 0.304); on the opposite, Mon2 CXCR4 expression (RFI) was significantly correlated with the number of calcified plaques (*P* = 0.0026, *R* = 0.345). TNF-*α* plasma levels exhibited positive correlations with number of mixed plus noncalcified lesions (*P* = 0.0362, *R* = 0.246) and alike IFN-*γ*/IL-10 cytokine ratio with that of mixed plaques (*P* = 0.0186, *R* = 0.275).

## 4. Discussion

The main findings of the present study are (1) a specific pro-inflammatory circulating monocyte phenotype is independently and significantly associated to more advanced CAD stages, from minimal to non-obstructive and to obstructive CAD and (2) this profile is partly related to a less calcified pattern of CTCA-assessed coronary plaques.

A previous retrospective cross-sectional study, performed in asymptomatic CAD patients, showed that only the count (n° of cells/*μ*l) of the intermediate CD14++/CD16+ monocyte subset was significantly associated with mixed and calcified plaque numbers and with stenosis severity [29]. In our study, we extended the multivariate regression adjustment to several clinical and immunological features known to influence phenotype and function of circulating monocytes, as well as their ability to adhere to activated endothelium and migrate into inflammatory sites and atherosclerotic plaques [1]. As a result, we have observed that not only a higher Mon2 subset circulating fraction but also a higher expression level of its activation markers (CD16), adhesion molecules (CD18), and chemokine receptors (CX3CR1) are independently correlated with more advanced CAD stage. The extended multivariate regression adjustment, as well as the CCR2-based flow cytometry quantification of circulating monocyte subsets performed in our study, could explain the differences observed with the previously cited study [29] regarding the associations between plaque composition and subset frequency and count. Interestingly, we also found that the increase of Mon2 subset frequency is related to the activation state (HLA-DR expression) of CD14++/+ cells, suggesting an accelerated activation-induced maturational transition of circulating mononuclear cells in patients with more severe disease. This hypothesis is also supported by the observation of independent correlations between stenosis severity and the expression level (as RFI) of monocyte activation markers HLA-DR and CX3CR1 in all CD14++/+ cell population. The inverse relationship of IL-10 plasma levels, as well as the direct relationship between increasing stenosis severity and IFN-*γ*/IL-10 cytokine ratio, further reinforce this hypothesis. Notably, stenosis severity and the expression level of fractalkine receptor CX3CR1, both on Mon2 subset and on all CD14++/+ mononuclear cells, are also significantly associated. This activation-induced chemokine receptor, in addition to playing a key role during monocyte migration, also regulates their survival and accumulation within inflamed tissues [30, 31].

Finally, our results indicate that the monocyte phenotypic pattern of more advanced CAD (higher Mon2 subset frequency and higher expression of some of its cell surface molecules, especially of the activation marker CD16) is also associated to a less calcified plaque pattern of the disease. In particular, the correlations found between the number of less calcified plaques and the CD16 RFI values, both on Mon2 subset and on all CD14++/+ monocyte, suggest that the expression level of this marker may represent a useful blood signature of a more unstable and inflammation-associated evolving atherosclerotic disease, albeit in the context of stable clinical conditions, as supported by the parallel increase of TNF-*α* plasma levels and IFN-*γ*/IL-10 cytokine ratio. On this basis, our findings confirm, and at the same time expand, previous acquisitions on the clinical utility of the Mon2 subset blood frequency in predicting plaque vulnerability and clinical outcome in asymptomatic patients [32, 33].

In summary, the results of our study indicate that, in stable CAD, the combination of a more advanced atherosclerotic disease by stenosis severity and a higher number of less calcified plaque lesions is reflected by specific changes of functional phenotype markers of the entire blood monocyte population and of its subsets, consistent with the existence of a pro-inflammatory systemic environment. In particular, deep phenotyping of circulating monocytes by flow cytometry assessment of Mon2 subset cell number and receptor expression emerges as a promising approach for monitoring CAD evolution in stable patients with chronic coronary syndrome.

The main purpose of this substudy of SMARTool clinical trial was to characterize the blood monocyte phenotype in stable CAD. The major limit is the relatively low number of patients included: the results of monocyte phenotypic association with CAD anatomical stage should be interpreted with caution. The need of processing immediately fresh blood for flow cytometry analysis, soon after coronary CT scan and inflammatory biomarkers assay is one of the main reasons for the low number of recruitments and for its limited use in clinical studies. For a clinical exploitation, observations on larger patient populations are needed to confirm pathophysiological hypotheses and associations between immunological features and the stenosis severity of CAD.

## Figures and Tables

**Table 1:** Demographic, laboratory and clinical characteristics of patients.

	All patients (*n*° = 73)	CAD1 (*n*° = 30)	CAD2 (*n*° = 21)	CAD3 (*n*° = 22)	ANOVA *P*
Demographics					
Age (years)	68.2 ± 0.9	65.48 ± 1.4	70.2 ± 1.7	69.7 ± 1.5	Ns
Sex (M/F, n°)	48/25	17/13	12/9	19/3	Ns
BMI (kg/m^2^)	27.36 ± 0.44	27.84 ± 0.63	26.77 ± 0.75	27.30 ± 0.98	Ns

Risk factors					
Framingham Risk Score (FRS)	15.32 ± 0.38	14.83 ± 0.76	16.10 ± 0.56	15.27 ± 0.62	Ns
Metabolic syndrome, *n*° (%)	2 (2.7)	1 (3.3)	0 (0.0)	1 (4.5)	Ns
Obesity, *n*° (%)	15 (20.5)	7 (23.3)	4 (19.0)	4 (18.1)	Ns
Hypertension, *n*° (%)	58 (79.4)	22 (73.3)	20 (95.2)	16 (72.7)	Ns
Active smoking, *n*° (%)	8 (10.9)	2 (6.6)	3 (14.2)	3 (13.6)	Ns
Dyslipidemia, *n*° (%)	65 (89.0)	24 (80.0)	20 (95.2)	21 (95.4)	Ns
Diabetes, *n*° (%)	21 (28.7)	4 (13.3)	6 (28.5)	11 (50.0)	0.0155
Family history of CAD, *n*° (%)	38 (52.0)	17 (56.6)	10 (47.6)	11 (50.0)	Ns

Laboratory exams					
WBC (10^9^/l)	7.91 ± 0.23	7.47 ± 0.35	8.57 ± 0.44	7.90 ± 0.43	Ns
Neutrophil (10^9^/l)	4.73 ± 0.19	4.48 ± 0.28	5.03 ± 0.38	4.79 ± 0.39	Ns
Monocyte (10^9^/l)	0.62 ± 0.02	0.58 ± 0.03	0.67 ± 0.04	0.63 ± 0.03	Ns
Lymphocyte (10^9^/l)	2.35 ± 0.08	2.19 ± 0.09	2.65 ± 0.16	2.29 ± 0.17	Ns
Platelet (10^9^/l)	226.13 ± 5.48	220.03 ± 7.55	237.09 ± 10.36	224.00 ± 11.34	Ns
Glucose (mg/dl)	102.54 ± 3.52	97.93 ± 2.93	105.67 ± 8.28	105.44 ± 7.44	Ns
Creatinine (mg/dl)	0.85 ± 0.02	0.87 ± 0.03	0.78 ± 0.04	0.90 ± 0.04	Ns
Acid uric (mg/dl)	5.34 ± 0.12	5.43 ± 0.20	5.31 ± 0.25	5.25 ± 0.20	Ns
Total cholesterol (mg/dl)	187.99 ± 4.46	197.57 ± 7.22	192.00 ± 8.94	171.09 ± 5.93	0.0373^∗^
HDL-cholesterol (mg/dl)	56.93 ± 1.98	60.40 ± 3.93	56.48 ± 2.41	52.64 ± 2.88	Ns
LDL-cholesterol (mg/dl)	104.78 ± 4.10	112.83 ± 6.11	109.55 ± 8.71	89.45 ± 6.09	0.0417^∗^
Triglycerides (mg/dl)	134.14 ± 7.38	121.50 ± 10.08	140.38 ± 12.00	145.41 ± 16.70	Ns
Hs-CRP (mg/dl)	0.38 ± 0.08	0.38 ± 0.10	0.33 ± 0.07	0.44 ± 0.20	Ns
Fibrinogen (mg/dl)	324.01 ± 8.96	324.18 ± 11.41	325.57 ± 19.51	322.29 ± 17.93	Ns
ICAM-1 (ng/ml)	214.35 ± 10.97	210.85 ± 15.15	222.04 ± 21.22	211.80 ± 22.86	Ns
VCAM-1 (ng/ml)	625.16 ± 17.95	652.58 ± 35.75	547.93 ± 17.11	661.48 ± 24.69	0.0210^§^^

Medications					
Statin therapy, *n*° (%)	50 (68.5)	15 (50.0)	14 (66.6)	21 (95.4)	0.0022
Statin therapy (dosage, mg/die)	11.5 ± 1.2	7.3 ± 1.5	13.1 ± 2.7	15.9 ± 2.1	0.0104^§^^∗^
Antihypertensive therapy, *n*° (%)	59 (80.8)	22 (73.3)	18 (85.7)	19 (86.3)	Ns
Insulin therapy, *n*° (%)	4 (5.4)	1 (3.3)	3 (14.2)	0 (0.0)	Ns
Oral hypoglycemic therapy, *n*° (%)	19 (26.0)	4 (21.0)	5 (26.3)	10 (52.6)	0.0321

Data are presented as mean ± SEM or as number (*n*°) and percentage (%), when appropriate. The Bonferroni post-hoc: ^∗^CAD1/CAD3, ^§^*CAD1/CAD2*, and ^*CAD2/CAD3*. Ns: not significant.

**Table 2:** Univariate and multivariate multinomial logistic regression analyses of blood monocyte subset frequency (%) and count (n° of cells/*μ*l).

Subset frequency and count	Comparison between groups	Univariate regression coefficient	Unadjusted OR	95% CI	*P*	Adjusted OR^a^	95% CI	*P*
Mon2 (%)	*CAD1/CAD2* *CAD1/CAD3*	0.156 0.193	1.169 1.213	0.978-1.398 1.017-1.447	Ns 0.0314	1.421 1.550	1.043-1.936 1.051-2.285	0.0260 0.0269
Mon3 (%)	*CAD1/CAD2* *CAD1/CAD3*	0.047 0.125	1.048 1.134	0.862-1.274 0.936-1.372	Ns Ns	1.351 1.355	0.885-2.061 0.865-2.125	Ns Ns
Mon1 (*n*°/*μ*l)	*CAD1/CAD2* *CAD1/CAD3*	0.003 0.001	1.003 1.001	0.999-1.006 0.996-1.005	Ns Ns	0.999 0.996	0.995-1.004 0.988-1.003	Ns Ns
Mon2 (*n*°/*μ*l)	*CAD1/CAD2* *CAD1/CAD3*	0.036 0.039	1.036 1.040	1.005-1.069 1.008-1.073	0.0237 0.0126	1.050 1.059	0.998-1.105 0.998-1.125	Ns Ns
Mon3 (*n*°/*μ*l)	*CAD1/CAD2* *CAD1/CAD3*	0.025 0.029	1.025 1.029	0.993-1.058 0.998-1.062	Ns Ns	1.031 1.014	0.974-1.091 0.961-1.070	Ns Ns

CAD: coronary artery disease; OR: odds ratio; CI: confidence interval; Ns: not significant. ^a^Adjusted for Framingham Risk Score (FRS), metabolic syndrome, diabetes, Hs-CRP, IL-6, IFN-*γ*, TNF-*α*, IL-10, VCAM-1, ICAM-1, use and dosage of statin therapy (mg/die), and use of oral hypoglycemic drugs.

**Table 3:** Univariate and multivariate multinomial logistic regression analyses of Mon2 markers (% positivity% and RFI).

Mon2 markers	Comparison between groups	Univariate regression coefficient	Unadjusted OR	95% CI	*P*	Adjusted OR^a^	95% CI	*P*
CX3CR1 (RFI)	*CAD1/CAD2* *CAD1/CAD3*	0.014 0.015	1.014 1.015	0.996-1.032 0.998-1.033	Ns Ns	1.055 1.057	1.009-1.103 1.008-1.108	0.0187 0.0215
CD18 (RFI)	*CAD1/CAD2* *CAD1/CAD3*	0.008 0.005	1.008 1.005	0.997-1.020 0.994-1.016	Ns Ns	1.030 1.028	1.005-1.055 1.002-1.054	0.0197 0.0338
HLA-DR (RFI)	*CAD1/CAD2* *CAD1/CAD3*	0.011 0.006	1.012 1.006	0.997-1.026 0.993-1.019	Ns Ns	1.028 1.029	0.998-1.059 0.999-1.060	Ns Ns
CXCR4 (RFI)	*CAD1/CAD2* *CAD1/CAD3*	0.011 0.013	1.011 1.013	0.997-1.025 0.999-1.027	Ns Ns	1.002 1.016	0.980-1.023 0.993-1.040	Ns Ns
CCR2 (RFI)	*CAD1/CAD2* *CAD1/CAD3*	0.014 0.005	1.014 1.005	1.001-1.027 0.992-1.017	0.0402 Ns	1.036 1.026	1.011-1.061 0.997-1.056	0.0042 Ns
CD163 (RFI)	*CAD1/CAD2* *CAD1/CAD3*	0.025 0.009	1.025 1.009	1.005-1.045 0.992-1.026	0.0126 Ns	1.037 1.031	1.004-1.071 0.993-1.069	0.0297 Ns
CD16 (%+)	*CAD1/CAD2* *CAD1/CAD3*	0.116 0.171	1.123 1.187	0.972-1.298 1.030-1.367	Ns 0.0179	1.341 1.463	1.021-1.761 1.051-2.037	0.0352 0.0240

CAD: coronary artery disease; OR: odds ratio; CI: confidence interval; Ns: not significant; %+: percentage of positivity; RFI: relative fluorescence intensity. ^a^ Adjusted for same variables of Table 3.

## Data Availability

The data used to support the findings of this study are available from the corresponding author upon reasonable request.

## Supplementary Materials

Supplementary Materials Supplemental Table S1: full list of inclusion, exclusion, and exit criteria of SMARTool clinical study. Supplemental Table S2: relationships between maximum stenosis classes and coronary artery plaque number.
