# Supplementary material for: Blood Monocyte Phenotype Fingerprint of Stable Coronary Artery Disease: A Cross-Sectional Substudy of SMARTool Clinical Trial
Source: Biomed Res Int. 2020 Jul 27;2020:8748934. doi: 10.1155/2020/8748934 (PMC7403909; doi:10.1155/2020/8748934)
Supplement: Supplementary Materials — Supplemental Table S1: full list of inclusion, exclusion, and exit criteria of SMARTool clinical study. Supplemental Table S2: relationships between maximum stenosis classes and coronary artery plaque number. [file 8748934.f1.doc]

Table S1

Full list of inclusion, exclusion and exit criteria of SMARTool Clinical Study

| **Inclusion criteria** |
| --- |
| 1) Male and female subjects, aged 48-82 years |
| 3) Caucasian population |
| 4) Submitted to CCTA for suspected CHD between 2009 and 2012 (in the context of EVINCI and ARTreat FPVII studies) at the Hospitals reported in “SMARTool Clinical Center” document and satisfying the eligibility criteria reported above |
| 5) Submitted to clinical follow-up in the last 6 months to verify stable clinical conditions and documented CHD or persistent intermediate/high probability of CHD |
| 6) Signed informed consents (clinical and genetic) |
| **Exclusion criteria** |
| 1) Multi-vessel severe disease (3 vessels and/or LM disease with >90% stenosis) |
| 2) Severe coronary calcification (CAC score > 600) |
| 3) Having undergone surgical procedures related to heart diseases (valve replacement, CRT or CRTD treatment, any surgery of the heart or arteries)  4) Documented MACE at history (myocardial infarction, severe heart failure, recurrent angina) in the last 6 months with/without revascularization |
| 5) Documented severe peripheral vascular disease (carotid, femoral) |
| 6) Surgery of carotid and/or peripheral arteries or cerebral ischemic attack |
| 7) History/surgery of Abdominal Aortic Aneurysm(AAA) |
| 8) Severe Heart failure (NYHA Class III-IV) |
| 9) LV dysfunction (left ventricle EF <40%) |
| 10) Atrial fibrillation |
| 11) Lack of written informed consent (clinical consent and/or genetic consent) |
| 12) Pregnancy (evaluated by urine test) and breastfeeding |
| 13) Active Cancer |
| 14) Asthma |
| 15) Cardiomyopathy or congenital heart disease |
| 16) Significant valvular disease (hemodynamically significant valvular stenosis or insufficiency by echoDoppler) |
| 17) Renal dysfunction (creatinine > 1.3 mg/dL) |
| 18) Chronic Kidney Disease (eGFR < 30 ml/min/1.73 m2) |
| 19) Hepatic failure (at least 3 of the following: albumin < 3.5 g/dL; prolonged prothrombin time–PT; jaundice; ascites) |
| 20) Waldenström disease |
| 21) Multiple myeloma |
| 22) Autoimmune/Acute inflammatory disease |
| 23) Previous severe adverse reaction to iodine contrast agent |
| 24) Positivity at blood tests for HIV, Hepatitis B and C (CRF number 1-clinical evaluation) |
| **Exit criteria** |
| A) Informed consent retired by the patient (genetic or clinical) |
| B) Adverse events to contrast medium during CCTA |

Table S2

*Stenosis based CAD-RADS severity classes and total number of plaques*

*detected by CTCA*

| N. pts | CAD-RADS severity class | Total n° of plaques detected by visual analysis (mean ± SD) |
| --- | --- | --- |
| 14 | 0 | 0 |
| 16 | 1 | 3.8 ± 3.0 |
| 21 | 2 | 5.3 ± 3.0 |
| 14 | 3 | 6.9 ± 2.6 |
| 8 | 4 | 7.5 ± 3.3 |

SD = standard deviation
